# Contrasting responses of photosynthesis and photochemical efficiency to ocean acidification under different light environments in a calcifying alga

**DOI:** 10.1038/s41598-019-40620-8

**Published:** 2019-03-08

**Authors:** Amy A. Briggs, Robert C. Carpenter

**Affiliations:** 1 Department of Biology, California State University, Northridge, Northridge, CA USA; 2 Odum School of Ecology, University of Georgia, Athens, GA USA

## Abstract

Ocean acidification (OA) is predicted to enhance photosynthesis in many marine taxa. However, photophysiology has multiple components that OA may affect differently, especially under different light environments, with potentially contrasting consequences for photosynthetic performance. Furthermore, because photosynthesis affects energetic budgets and internal acid-base dynamics, changes in it due to OA or light could mediate the sensitivity of other biological processes to OA (e.g. respiration and calcification). To better understand these effects, we conducted experiments on *Porolithon onkodes*, a common crustose coralline alga in Pacific coral reefs, crossing pCO_2_ and light treatments. Results indicate OA inhibited some aspects of photophysiology (maximum photochemical efficiency), facilitated others (α, the responsiveness of photosynthesis to sub-saturating light), and had no effect on others (maximum gross photosynthesis), with the first two effects depending on treatment light level. Light also exacerbated the increase in dark-adapted respiration under OA, but did not alter the decline in calcification. Light-adapted respiration did not respond to OA, potentially due to indirect effects of photosynthesis. Combined, results indicate OA will interact with light to alter energetic budgets and potentially resource allocation among photosynthetic processes in *P. onkodes*, likely shifting its light tolerance, and constraining it to a narrower range of light environments.

## Introduction

Ocean acidification (OA), which decreases seawater pH and shifts its carbonate chemistry, has significant consequences for the physiology of calcifying photosynthetic taxa^1–3^. OA generally decreases calcification rates^3^, primarily due to the increased difficulty of building and maintaining calcium carbonate structures under these conditions. OA also affects photophysiology, although the direction and magnitude of these effects vary across taxa^3,4^ and the aspect of photophysiology being considered, leading to different consequences for photosynthetic performance under different environmental contexts.

For example, photosynthesis may increase under OA in some species as carbon acquisition becomes easier for them due to the increased availability of dissolved inorganic carbon (DIC)^5–7^. However, this effect would be expected only under conditions when photosynthesis is not limited by other factors such as light. Additionally, for species that use carbon-concentrating mechanisms (CCMs) to reduce carbon limitation, OA could increase the energetic efficiency of carbon acquisition, by allowing these species to down-regulate energetically expensive carbon-concentrating enzymes^8^. In this case, these taxa might not see an increase in their rate of photosynthesis with OA (depending on their type of CCM and their degree of carbon limitation in contemporary environments)^4^, although energetic savings associated with easier carbon acquisition under OA could facilitate other biological processes.

In contrast to these potential benefits to carbon acquisition, OA may negatively affect other aspects of photophysiology. Specifically, the expression of specific proteins and/or maintenance of enzymes related to photophysiology may be altered by OA, which has been shown to alter protein metabolism and accumulation in a variety of photosynthetic^9^ and non-photosynthetic taxa^10^. These effects may not influence metabolic rates like photosynthesis, but could have significant consequences at the cellular level, affecting resource allocation among biological processes and potentially leading to shifts in organismal performance under certain environmental conditions. In the case of photosynthesis, protein turnover is particularly important for repairing damage in Photosystem II (PSII) caused by light^11,12^. This damage can reduce the photochemical efficiency of PSII, eventually causing photoinhibition (i.e. a reduction in photosynthetic capacity associated with light stress) when damage is large enough in magnitude and duration^13,14^. Thus, modified or impaired protein metabolism could influence enzymes related PSII function, with increasingly detrimental effects on photochemical efficiency as light increases (and damage to PSII is elevated). Additionally, under low light conditions, photoacclimation processes that facilitate light harvesting also require protein production and maintenance. Therefore, changes in protein metabolism due to OA could enhance negative effects of both high and low light on photophysiology.

Increases in either the rate of photosynthesis or in its efficiency (either due to the effects of OA or other environmental factors such as light) could reduce the sensitivity of other physiological processes like calcification and respiration to OA. For example, enhanced photosynthesis (either in terms of the energetic efficiency of carbon fixation or in its overall rate) could provide additional energy to perform metabolic work to compensate for the effects of OA on these processes. Additionally, hydroxide ion production by photosynthesis^15,16^ may facilitate calcification by increasing calcification site pH^15,17^, as well pH within the diffusion boundary layer surrounding the organism, (the latter of which likely reduces dissolution of calcified structure during the daytime)^16,18^. These effects on internal acid-base dynamics could also save energy by reducing the need for active processes that maintain pH and charge balance in intracellular spaces. Some of these processes, such as proton export, should be more costly under OA, due to elevated proton concentrations in the surrounding seawater^19^. Therefore, an organism that photosynthesizes faster could hypothetically see less of a reduction in its calcification rate and/or less of an increase in its respiration under OA relative to one with a lower photosynthetic rate.

One group of calcifying phototrophs that will likely be affected by OA are crustose coralline algae (CCA). CCA are typically sensitive to OA, showing reduced calcification^20–22^ and recruitment^23^ under acidified conditions. These negative effects may have important consequences in habitats where CCA are found, particularly in coral reef ecosystems, where CCA are important agents of reef accretion, and also influence recruitment of benthic invertebrates like corals^24,25^. Coralline red algae, including CCA, have carbon-concentrating mechanisms (CCMs)^5,16^. However, not enough is known about these mechanisms to determine whether or not OA should release CCA from carbon limitation, or if their CCM expression is flexible enough to lead to energetic savings under OA.

To gain a better understanding of how OA affects photophysiology in CCA under different light environments, and potential cascading effects on the responses of calcification and respiration to OA, we conducted two laboratory experiments in Moorea, French Polynesia, on *Porolithon onkodes*, a widespread tropical Pacific species of CCA. However, due to issues with equipment and logistical limitations, the first experiment became a pilot study that only tested a subset of our research questions. Because of this, we focus here on the second experiment, but include the pilot study in Supplementary Material S1 to demonstrate its qualitatively similar results. In both experiments, we crossed two pCO_2_ and three light treatments, and measured a variety of physiological responses to test the following hypotheses: 1.) OA affects the rate of photosynthesis, but also influences PSII function, and these effects are modulated by light. Specifically, stimulation of photosynthesis can occur under OA due to increased carbon availability, but only at light levels that are saturating for photosynthesis. Additionally, OA negatively affects photochemical efficiency of PSII, and these effects are magnified under high light conditions. 2.) Photosynthesis can mitigate some of the effects of OA on respiration and calcification, and this effect is modulated by light, due to the influence of light on the rate of photosynthesis. Thus, we predicted that *P. onkodes* in higher light conditions would have more rapid photosynthesis, and would show less of a reduction in calcification under OA, and less of an increase in respiration, particularly if this increase in photosynthesis was greatest in the high light, high pCO_2_ (i.e. OA) treatment.

## Results

### Physical Parameters of the Experiment

Mean light and pCO_2_ treatments were maintained at relatively consistent values within the experiment, as were other physical parameters such as temperature and total alkalinity (summarized in Supplementary Table S3). pCO_2_ was 406.1 ± 2.3 SEM µatm in the ambient treatment and 995.2 ± 15.0 SEM µatm in the elevated treatment. Mean light levels were 26, 67, and 413 μmol photons m^−2^ s^−1^ in the low, medium, and high light treatments respectively. (See Supplementary Table S3 for means with error by light x pCO_2_ group.) The high light treatment was above the light saturation point for photosynthesis in this species (See the *P*-*E* curve results below).

### Photosynthesis

**Figure 1:**
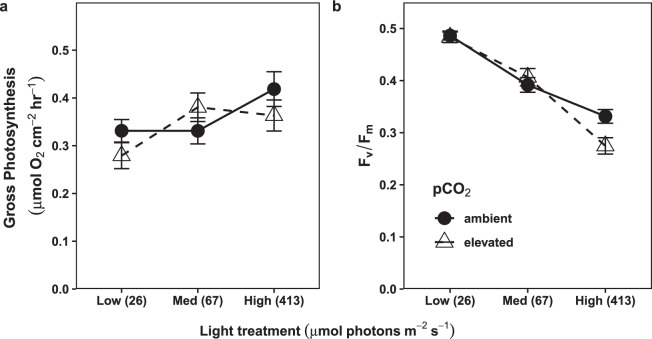
Photophysiological responses of CCA to experimental OA and light treatments. Points represent group means ± SEM. The x-axis indicates experimental light treatments, with the mean photon flux density (PFD; µmol photons m^-2^ s^-1^) for each treatment in parentheses. (**a**) Area-normalized gross photosynthesis rates, measured at approximately the same PFD that the CCA were kept under in their experimental light treatments (n = 16). Gross photosynthesis for each CCA sample was calculated as the sum of its rate of O_2_ evolution through net photosynthesis, plus its rate of O_2_ consumption through light-adapted respiration. Photosynthesis increased significantly with treatment light level (p = 0.016), but did not respond to pCO_2_. There was no significant light x pCO_2_ interaction. (**b**) Maximum photochemical efficiency (F_v_/F_m_) of dark-adapted samples (n = 32). There was a significant interaction between light and pCO_2_ (p = 0.016).

Gross photosynthesis increased significantly with light (p = 0.02), increasing on average by 27% between the low and high light treatments. However, pCO_2_ did not affect gross photosynthesis, nor did the interaction between pCO_2_ and light (Fig. 1a). All test statistics and p-values are summarized in Supplementary Table S4.

**Figure 2:**
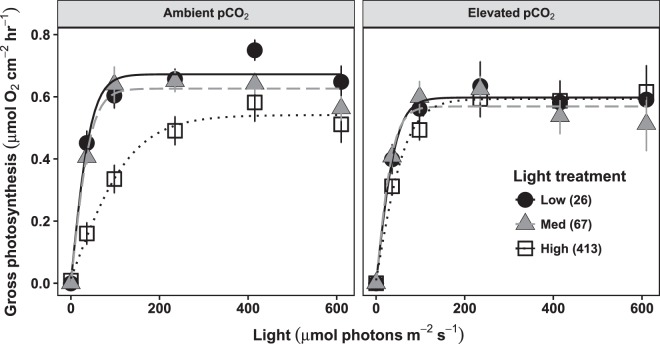
Photosynthesis-light response (*P*-*E*) curves, measured using the change in O_2_ concentrations at six photon flux densities (PFDs, µmol photons m^−2^ s^−1^). Points represent mean gross photosynthesis rates (net photosynthesis + dark-adapted respiration) for each pCO_2_ and light treatment group (n = 8) at a given PFD. Error bars represent SEM, with data fitted by a hyperbolic tangent function. Shapes represent the light treatments that samples were kept under in their experimental tanks, with the mean PFD for each treatment indicated in parentheses in the figure legend.

In the photosynthesis-light response (*P*-*E*) curves constructed from a subset of CCA from each light x pCO_2_ treatment, photosynthesis increased rapidly with light, and reached light saturation before 200 µmol photons m^−2^ s^−1^ (Table 1), indicating the high light treatment was saturating for photosynthesis (Fig. 2). The maximum rate of gross photosynthesis, *P*_*max*_, was not affected significantly by pCO_2_ or by light, or their interaction, and across all treatments had a mean of 0.60 μmol O_2_ cm^−2^ hr^−1^ ± 0.01 SE. However, the initial slope of the *P*-*E* curves (*α*) demonstrated a significant interaction between pCO_2_ and light (p = 0.05), such that the decrease in *α* as treatment light level increased did not occur under elevated pCO_2_ (Fig. 2). Due to this change, *α* was on average 56% lower under ambient pCO_2_ compared to elevated in the high light treatment (Table 1). In the medium and low light treatments, *α* was similar across pCO_2_ levels, averaging 0.014 μmol O_2_ cm^−2^ hr^−1^/μmol photons m^−2^ s^−1^.

**Table 1:** Summary of the photosynthesis-light response (*P*-*E*) curve parameters for each treatment, with the additional parameter *E*_*k*_ (the light saturation point), calculated as *P*_*max*_/*α*.

*P*-*E* curve parameter	Treatment					
	ACO_2_-HL	HCO_2_-HL	ACO_2_-ML	HCO_2_-ML	ACO_2_-LL	HCO_2_-LL
Initial slope (*α*)	0.004 ± 0.001, a	0.009 ± 0.001, ab	0.013 ± 0.002, b	0.015 ± 0.002, ab	0.015 ± 0.002, b	0.013 ± 0.002, ab
Max. gross photosynthesis (*P*_*max*_)	0.544 ± 0.053	0.598 ± 0.055	0.628 ± 0.035	0.587 ± 0.052	0.670 ± 0.020	0.605 ± 0.064
Saturating light (*E*_*k*_)	161.3 ± 22.6	90.5 ± 24.9	56.7 ± 11.5	52.8 ± 16.3	50.7 ± 7.0	55.0 ± 7.4

Values represent treatment means ± SEM (n = 8). Treatments abbreviations are as follows— ambient pCO_2_ (ACO_2_); elevated, i.e. high, pCO_2_ (HCO_2_); low (LL), medium (ML), and high (HL) light. Units are μmol O_2_ cm^−2^ hr^−1^/μmol photons m^−2^ s^−1^ for *α*, μmol O_2_ cm^−2^ hr^−1^ for *P*_*max*_, and μmol photons m^−2^ s^−1^ for *E*_*k*_. Post hoc tests were conducted for *α*, as this parameter had a significant light x pCO_2_ interaction. Groups sharing the same letter are not significantly different (alpha = 0.05, Tukey-adjusted).

### Photochemical efficiency

Overall, maximum photochemical efficiency (F_v_/F_m_) of dark-adapted samples declined as the treatment light level increased (from 0.49 to 0.33 under ambient pCO_2_, and 0.48 to 0.27 under elevated pCO_2_)_._ However, there was a significant interactive effect of light and OA (p = 0.016), such that OA had a stronger negative effect on F_v_/F_m_ in higher light treatments (Fig. 1b). In the highest light treatment, this interaction resulted in an average F_v_/F_m_ that was 17% lower in the OA treatment compared to ambient.

### Respiration

**Figure 3:**
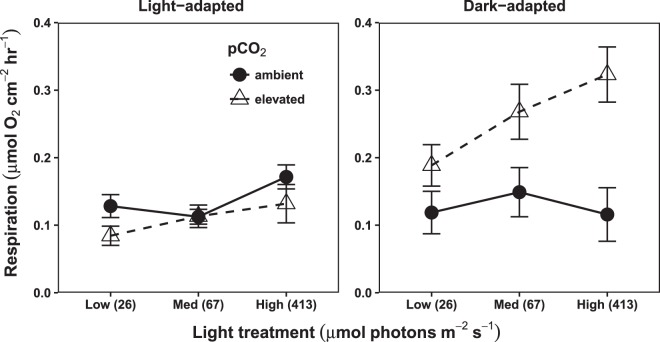
Light vs. dark-adapted respiration. Points represent treatment group means ± SEM. Rates are in terms of O_2_ consumed, normalized to the surface area of the algal samples (n = 16 for light-adapted respiration, and n = 8 for dark-adapted respiration). The x-axis indicates experimental light treatments, with the mean photon flux density (µmol photons m^−2^ s^−1^) for each treatment in parentheses. Light-adapted respiration increased with treatment light level (p = 0.007). pCO_2_ did not affect light-adapted respiration. Dark-adapted respiration had a significant light x pCO_2_ interaction (p = 0.039).

Light-adapted respiration increased significantly with light (p < 0.01), increasing by 43% between the low and high light treatments (averaged across pCO_2_ treatments). However, it was not affected by pCO_2_, nor was there a significant interaction between pCO_2_ and light. In contrast, dark-adapted respiration was affected by the interaction between pCO_2_ and light (p = 0.04), such that rates were higher under elevated pCO_2_ compared to ambient. The magnitude of this difference increased with treatment light level (Fig. 3).

### Calcification

**Figure 4:**
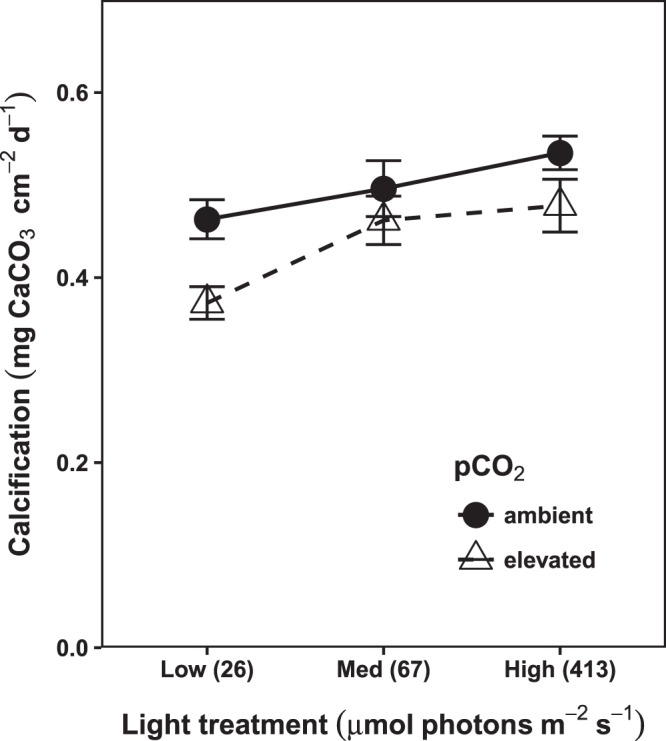
Area-normalized net calcification rates of *P. onkodes*. Points represent treatment group means ± SEM (n = 32). The x-axis indicates experimental light treatments, with the mean photon flux density (µmol photons m^−2^ s^−1^) for each treatment in parentheses. Calcification declined significantly under elevated pCO_2_ (p = 0.046), and increased with light (p = 0.001), but there was no light x pCO_2_ interaction.

Net calcification was positively correlated with light (p = 0.001), increasing by 22% in the high light treatment relative to the low light treatment. In contrast, calcification significantly decreased under elevated pCO_2_ (p = 0.046), declining by 15% relative to ambient (Fig. 4). There was no significant interaction between pCO_2_ and light (Supplementary Table S4).

## Discussion

Our results indicate increased carbon availability under OA does not directly stimulate photosynthesis in this species: neither *P*_*max*_, nor gross photosynthesis in the highest light treatment (which were at or above the light saturation point (*E*_*k*_) for *P. onkodes*^26,27^, Table 1) increased in the elevated pCO_2_ treatment. This result suggests that this species has a CCM with high affinity for DIC, preventing carbon limitation under ambient conditions. However, another (non-mutually exclusive) explanation for this result is that any potential benefit to photosynthesis caused by increased carbon availability is counterbalanced by additional effects of OA on other aspects of physiology in this species.

The results for maximum photochemical efficiency (F_v_/F_m_) support this latter hypothesis. F_v_/F_m_ is commonly used to estimate stress within PSII^14^, and a change in it under OA would imply that OA altered cellular and/or biochemical processes related to PSII function. Like we observed in our study, F_v_/F_m_ typically declines for organisms found in higher light environments, which is an acclimatization response that protects their photosynthetic machinery from light damage^28,29^. Such a decline does not necessarily result in a decreased rate of gross photosynthesis, unless the decline is large enough in magnitude and duration^13,14^. However, the increasingly negative effect of OA on F_v_/F_m_ as treatment light level increased indicates that OA exacerbates its decline with light, potentially by reducing the repair rate of light-damaged proteins (e.g.^11,12^). Studies on phytoplankton have found similar synergistic effects of light and OA on other aspects of PSII function, including non-photochemical quenching, another indicator of light stress^30–32^. Therefore, effects of OA on PSII function may be an important mechanism through which it alters photophysiology across multiple taxa.

Another indicator that OA has important indirect effects on photophysiology was the interactive effect of light and OA on *α*, the responsiveness of photosynthesis to sub-saturating light. Generally, *α* was negatively correlated with treatment light level, another photoacclimation phenomenon that allows phototrophs to optimize their energy accrual. However, in the highest (saturating) light treatment, *α* was over two times higher under OA conditions (Table 1). This result implies that in *P. onkodes* acclimatized to saturating light regimes, OA causes photosynthesis to increase more rapidly as light increases (until the light saturation point). Potentially tradeoffs between carbon acquisition and light harvesting, which has been observed indirectly in other red algae^33^, could explain this result. Specifically, reduced carbon capture and assimilation costs under OA could make more energy available for processes associated with light harvesting, such as the production of antennae pigments (components of the photosynthetic light harvesting complex attached to the chlorophyll reaction center), which could lead to an increase in *α*^34^. The interactive effect of light and OA on *α* may occur because both light and OA influence the production and activity of CCMs in algae—generally, CCMs are down-regulated under lower light levels as well as under OA^7,35^. Therefore, CCA in the low and medium light treatments potentially had low CCM production, and thus in these light treatments there was negligible down-regulation of CCM activity in the OA treatment relative to ambient seawater conditions. In contrast, in the high light treatment, reduced CCM activity associated with the OA treatment could free up resources for increased pigment production, thereby enhancing photosynthesis at sub-saturating light levels (and leading to a higher *α*).

This hypothesized tradeoff between CCM production and antennae pigment production under different light and pCO_2_ environments is consistent with our finding that OA had an increasingly negative effect on F_v_/F_m_ as treatment light level increased. Larger antenna size is believed to increase the susceptibility of PSII to photoinhibition^36^, and this susceptibility is frequently inferred from declines in F_v_/F_m_^37,38^. Thus, increased antennae size could have facilitated photosynthesis at sub-saturating light levels in the *P*-*E* curve measurements, leading to an increase in *α*, while also increasing susceptibility to photoinhibition (and decreasing F_v_/F_m_) in the saturating light treatment. Furthermore, internal CCMs have been implicated as a method for the dissipation of excess excitation energy^39–41^, which could help reduce photoinhibition at high light levels. Thus, decreased production of CCMs in high light conditions under OA may lead to an increased susceptibility of PSII to photoinhibition, which again is consistent with our findings for F_v_/F_m_. These potential linkages between carbon concentrating mechanisms, light harvesting pigments, and the effects of OA on photochemical efficiency and the photosynthesis-light relationship require further investigation. However, if accurate, they imply that the indirect effects of OA – including shifts in the expression or function of specific proteins related to photophysiology – may be just as important in determining the responses of marine phototrophs to OA as its direct effects on their carbon capture efficiency and rates of photosynthesis.

Regardless of the mechanisms, results from this study indicate that OA inhibits some aspects of photophysiology in *P. onkodes* (F_v_/F_m_), but also has neutral (*P*_*max*_) and facilitative effects on other aspects (e.g., *α*), and these effects are modulated by the light environment that the organism is acclimatized to. These contrasting results emphasize that it is not appropriate to assume that the response of one metric of photosynthetic performance will be representative of the response of another to OA. Furthermore, important patterns might be obscured if multiple metrics are lumped together for synthetic analyses of the effects of OA on photosynthesis (as has been done in previous meta-analyses and reviews).

In addition to these mixed responses by different components of photophysiology to OA and light, we found that dark-adapted and light-adapted respiration also showed different patterns from one another (Fig. 3). Dark-adapted respiration was higher in the OA treatment, with this difference increasing with treatment light level. Several studies on phytoplankton^31,42^ and photosynthetic foraminifera^43^ have found similar increases in dark-adapted respiration under OA, indicating a common effect across multiple photosynthetic taxa. However, light-adapted respiration did not show an effect of OA (consistent with findings in other studies on calcifying macroalgae^20^). This contrast with dark-adapted respiration suggests that changes in biochemical or physiological processes in the light vs. dark, such as hydroxide production by photosynthesis^15^ or proton pumping^16^, could modulate the response of respiration to OA. However, the magnitudes of the effects of these processes are not necessarily determined by the rate of photosynthesis (which increased with treatment light level, Fig. 1a). More work will be necessary to determine the mechanisms behind these differing responses. However, our results indicate that estimates of the effect of OA on respiration integrated over a daily cycle of light and dark could be biased in studies where it is measured under a single set of conditions (light or dark-adapted).

Similar to the light-adapted respiration results, the effect of OA on net calcification was not modified by light environment. Rather, in both experiments calcification increased with treatment light level, but consistently declined under OA and (Fig. 4), which agrees with previous findings in CCA^44,45^. Thus, we can infer that increased gross photosynthesis under higher light treatments was not sufficient to reduce the negative effects of OA on calcification. Potentially an ameliorative effect of photosynthesis would have been possible if there had been a greater relative increase in it under the high light-high pCO_2_ treatment. However, work in CCA has described sigmoidal, saturating relationships between calcification and photosynthesis^46^, and thus there simply may be limits to the ability of photosynthesis to stimulate calcification in this species. Furthermore, since light has an asymptotic relationship with calcification as well as photosynthesis^47^, there are also likely limits to its effects as well.

In other calcifying phototrophs such as corals, beneficial effects of light are possible when light is above the saturation point for calcification^48^. However, negative effects of high light have also been observed^49^, and might occur when light is high enough to induce photoinhibition and/or bleaching, which can reduce the amount of tissue actively calcifying. OA-induced bleaching occurs in *P. onkodes* under higher photon flux densities (700–1000 µmol photons m^−2^ s^−1^) than those used in this experiment^21,50^. This evidence, along with our finding that the negative effect of OA on photochemical efficiency increases with light (Fig. 1b), suggests that photoinhibition or bleaching could occur under OA in *P. onkodes* populations found in very shallow, high light habitats (which can reach 1500–2000 µmol photons m^−2^ s^−1^ ^26,51^. These effects could further reduce calcification, especially as these shallow environments also receive ultraviolet radiation, which amplifies the negative effects of OA on photosynthesis and calcification in coralline algae^52^.

Overall, our results indicate that OA will likely alter energetic budgets and potentially resource allocation in *P. onkodes*, and that these effects will vary across different light environments. Increases in respiration at night will increase the minimum amount of light that *P. onkodes* requires over a day to maintain a positive energetic balance, while increases in *α* for individuals found in brighter light environments may help offset increased respiration costs. Additionally, alterations to energy allocation between carbon capture and light harvesting may alter other facets of this organism’s biology such as its PSII function, potentially reducing the upper end of its light tolerance. Combined, these results indicate OA could constrain *P. onkodes* to a narrower range of depths and light microhabitats—a goldilocks area that is not too bright, due to the enhanced negative effects of high light on photochemical efficiency, and not too dark, due to the increased energetic needs associated with increased rates of respiration at night. Since *P. onkodes* often forms shallow ridges along reef crests^53^ that protect inner reefs and near-shore human populations, and is also a competitively dominant CCA species in shallow Indo Pacific reefs^54^, a shift in its distribution across reef habitats, particularly out of shallow, high light areas, could lead to cascading changes in the physical and community structure of many coral reef ecosystems.

## Methods

### Experimental setup

A laboratory experiment was conducted at the Richard B. Gump South Pacific Research Station on the island of Moorea, French Polynesia during June-July in 2014. Individual *Porolithon onkodes* crusts without obvious epibionts were collected using a hammer and chisel at a depth of 1–2.5 m, approximately 100–200 m from reef crest on the back reef on the North shore of Moorea, near Cook’s Bay (17°28′40.51″S, 149°50′22.80″W). See Supplementary Material S1 for more information on CCA species identification. Although some individuals of this species were found in semi-cryptic reef environments, experimental specimens were collected from open reef substrates, which had a mean photon flux density of 832 ± 134 (SD) µmol photons m^−2^ s^−1^ at daily peak solar irradiance (within an hour of local apparent noon; methods in Supplementary Material S1). CCA crusts (n = 192) were brought to the lab and trimmed to similar sizes (~ 9 cm^2^), and any visible bioeroding organisms (e.g., sponges, worms, etc.) were removed from their undersides before the exposed calcium carbonate skeleton was sealed using marine epoxy (Z-SPAR, Splash Zone). Following this procedure, samples were placed in a high flow acclimation tank for 9 days to heal and photoacclimate to the treatment light levels (26, 67, 413 µmol photons m^−2^ s^−1^). Previous work has found that photoacclimation processes generally occur within this time frame in red algae^55,56^. After the acclimation period, samples were placed into one of eight 150-L tanks under their randomly assigned pCO_2_-light treatments. Within each tank there were three light treatments constructed from acrylic frames topped with neutral-density filters, which manipulated light levels to the desired photon flux density. Photosynthesis exhibits a nonlinear relationship with light^47^, increasing up to a saturating light level, after which it becomes limited by other factors. Thus, if OA directly stimulated photosynthesis by releasing it from carbon limitation, we would expect to see photosynthesis increase after this light saturation point. We chose three treatment light levels to represent different points along the photosynthesis-light response (*P*-*E*) curve for *P. onkodes*, based on previous work on this species conducted on individuals from a similar range of depths and light environments as in those used in this study^26,27^. Low and medium light treatments (26 and 67 µmol photons m^−2^ s^−1^) represented points along the light-limited portion of the curve for this species, whereas the highest light treatment (413 µmol photons m^−2^ s^−1^) was chosen to fall above the light-saturation point of the curve, which was confirmed during the *P*-*E* curve measurements. To minimize any positioning effects within the tanks, samples were rotated underneath their light treatments every fourth day, and were brushed gently with a soft-bristle toothbrush to prevent turf algae or biofilm from establishing on their surface.

Four of the tanks were kept at ambient pCO_2_ (~406 µatm), and four were maintained at an elevated pCO_2_ (~995 µatm), by bubbling CO_2_-enriched air into them using a solenoid-controlled gas regulation system (Model A352, Qubit Systems). The high pCO_2_ treatment value was chosen to approximate predictions for the year 2100 under the IPCC’s Representative Concentration Pathway (RCP), Scenario 8.5^57^. Water in the tanks was replaced using filtered seawater from Cook’s Bay at a mean rate of 6.6 L hr^−1^, and tank temperature was maintained at 27 °C, a typical temperature for the Moorea backreef where the CCA were collected during the season in which the experiments were conducted^58^. The tanks were illuminated with LED lights (Aqua Illumination, Sol), which were kept on a 12-hour ramping light/dark cycle. PFDs were measured under light treatments in each tank once each week throughout the experiment during the period of peak irradiance using a 2π PAR sensor attached to a diving-PAM (Walz, Germany), calibrated to a LiCOR 2π PAR sensor (LI-1400).

### Seawater chemistry

Temperature was measured twice daily (07:30 and 17:30 hr) using a portable temperature probe (Fisher-Scientific, USA), and tank pH (total scale) was measured daily at 07:30 hr from water samples collected from each tank, using a titrator pH probe (Mettler-Toledo, DGi115-SC) that was calibrated every other day using a TRIS buffer solution. Total alkalinity (TA) was measured for these water samples every other day using an automatic open-cell potentiometric titrator (Mettler-Toledo, T50). Accuracy of the titrations was measured by titrating certified reference material from the Dickson lab (University of California, San Diego). Accuracy was ± 9.7 ± 1.3 μmol kg^−1^ (n = 21). Tank salinity was measured every other day using a conductivity meter (YSI 3100, YSI, USA). Carbonate chemistry of the tanks then was calculated from their salinity, temperature, pH, and TA values using the *seacarb* package in R^59^. Carbonate chemistry and other physical conditions for the treatments are summarized in Supplementary Table S3.

### Calcification

Net calcification rates of the CCA over the course of the experiment were calculated from differences in the buoyant weight of each sample measured at the beginning and end of the experiment^60^. Buoyant weights were converted to skeletal dry weights using the density of calcite (2.71 g cm^−3^). Although high-Mg calcite is the most common crystalline structure of calcium carbonate produced by CCA^61^, calculating its exact density requires knowing the mol % of Mg, which can vary due to a variety of factors^62^. Since the mol % of Mg was not known for these CCA, the density of calcite was used to estimate skeletal dry weight. The change in skeletal dry weight was calculated for each sample, and then was normalized to the duration of the experiment (28 days), as well as the surface area of the sample. Surface area of the living tissue of each sample was estimated using the foil method^63^.

### Photosynthesis and Respiration

Net photosynthesis and respiration rates were measured as the change in dissolved oxygen concentration in closed respirometry chambers (250 mL). This was done for a subset of CCA from each treatment group (n = 16 for photosynthesis and light-adapted respiration, and n = 8 for dark-adapted respiration). Oxygen concentrations were measured using a 2-mm diameter optical oxygen probe (PreSens, DP-PSt3) and logging system (PreSens, Precision Sensing GmbH, Germany). Incubations lasted 25–50 minutes, and were made at the same temperature and pCO_2_ that the samples experienced in the tanks, and at approximately the same light levels for the photosynthesis measurements (28, 69, and 416 µmol photons m^−2^ s^−1^). See Supplementary Material S1 for a full description of methods for incubations. Post-illumination (i.e., light-adapted) respiration was measured immediately after net photosynthesis by fully darkening the incubation chambers and measuring O_2_ consumption rates. Dark-adapted respiration was measured after the alga had been dark*-*acclimated for 45–60 minutes to minimize the stimulatory effect of photosynthesis on respiration^64^. The incubations for dark-adapted respiration took place as part of the *P*-*E* curve measurements (described in the following section). Respiration rates of control seawater samples taken from each of the experimental tanks were measured to isolate any signature of microbial activity within the tanks. The control samples all demonstrated a small respiration signal (i.e. a decline in O_2_ concentrations), so the absolute value of these negative rates were added to the photosynthesis and respiration rates measured for samples from the same tank. Gross photosynthesis was calculated for each CCA from its net photosynthesis rate plus the absolute value of the light-adapted respiration rate. Photosynthesis and respiration rates were normalized to the surface area of the algal sample.

### Photosynthesis-light response (P-E) curves

In addition to measuring net photosynthetic rates of the CCA at their treatment light levels, net photosynthesis was measured at six increasing PFDs (0, 36, 98, 235, 415, 610 µmol photons m^−2^ s^−1^) for a separate subset of samples (n = 8 per treatment). Rates were measured using the respirometry methods described above (with additional details in Supplementary Material S1). These rates were used to construct photosynthesis-light response (*P*-*E*) curves for individual samples. The *P*-*E* data were fit with a hyperbolic tangent function^47^ (Equation 1).

1 $$P{(E)}_{net}={P}_{max}\,\tanh (\frac{\alpha E}{{P}_{max}})+{P}_{0}$$

This model uses the incident photon flux density (*E*) and the measured rate of net photosynthesis at *E*, to determine *α*, the initial slope of the light-limited part of the curve (or the responsiveness of photosynthesis to sub-saturating light) and *P*_*max*,_ the maximum gross photosynthetic rate. *P*_0_ is the rate of O_2_ consumption when *E* = 0, i.e., the dark-adapted respiration rate.

### Photochemical Efficiency

A diving-PAM (pulse-amplitude modulated fluorometer, Walz GmbH, Germany) was used to estimate the maximum photochemical efficiency of PSII^13,62^ of dark-adapted samples (F_v_/F_m_, n = 32). See Supplementary Material S1 for details.

### Statistical analyses

Because many of the response variables are thought to be strongly correlated with each other, we performed separate analysis of variance (ANOVA) on each variable to test the effects of pCO_2_ and light. These variables included net calcification, respiration, photosynthesis, and photochemical efficiency, as well as the parameters *α* and *P*_*max*_ estimated using the *nls()* function in R for the individual sample *P*-*E* curves. Additionally, since the light and pCO_2_ treatments were slightly different for the two experiments, we analyzed results separately for each experiment. For each analysis, a mixed-effects model was constructed with light and pCO_2_ treatments as fixed factors and tank as a random factor, using the R package *nlme*^65^. Model residuals were checked graphically for heteroscedasticity, in addition to statistically testing for homogeneity of variances using Levene’s test (using the *car* package in R^66^), and all variables met the assumptions for ANOVA. Tukey *post hoc* tests were conducted on *P*-*E* curve parameters that exhibited significant main effects or interactions to determine which groups were different from one another. Tukey adjustments were used to correct p-values for multiple comparisons in the *post hoc* tests. All statistical analyses were done using R 3.3.2^67^.

## Supplementary information

Supplementary Material S1

## Data Availability

The data generated during this study are available in the Moorea Coral Reef LTER dataset repository at http://mcr.lternet.edu/data.
