# Supplementary material for: Contrasting responses of photosynthesis and photochemical efficiency to ocean acidification under different light environments in a calcifying alga
Source: Sci Rep. 2019 Mar 8;9:3986. doi: 10.1038/s41598-019-40620-8 (PMC6408467; doi:10.1038/s41598-019-40620-8)
Supplement: Supplementary file 1 — Supplementary Material S1 [file 41598_2019_40620_MOESM1_ESM.pdf]

Supplement S1

Contrasting responses of photosynthesis and photochemical efficiency to ocean acidification under different light environments in a calcifying alga

Amy A. Briggs, Robert C. Carpenter

Scientific Reports. 2019.

Pilot study description and results, and comparison with the 2014 experiment

Methods

In July 2013, a pilot study was conducted at the Richard B. Gump South Pacific Research Station on the island of Moorea, French Polynesia, to explore how light modifies the effects of OA on calcification, respiration, and photosynthesis in *Porolithon onkodes*. Unless otherwise noted, all methods, calculations, and equipment for this study were the same as those used in the 2014 experiment. *P. onkodes* samples (n = 240) were collected from the same backreef site on the North shore of Moorea, and were prepared and acclimated to experimental light levels for 10 days before being transferred to the experimental tanks.

Four 150-L tanks were kept at ambient pCO_2 (~ 380 µatm), and four were maintained at an elevated pCO_2 (~ 833 µatm), with three light treatments (23, 55, and 210 µmol photons m^{-2} s^{-1}) within each tank. Lighting equipment was the same in both experiments, but was refurbished before the 2014 experiment so that we could achieve a higher high-light treatment. Water in the tanks was replaced using filtered seawater from Cook's Bay at a mean rate of 8.7 L hr^{-1}, and tank temperature was maintained at 27 °C. Tank conditions (temperature, light, pH, salinity, total alkalinity) were measured using the previously mentioned procedures, although total alkalinity was initially measured daily instead of every other day. Total alkalinity measurements had an accuracy of 4.5 ± 0.6 (SEM) µmol kg^{-1} (n = 20). Experimental conditions and carbonate chemistry of each treatment are summarized in Table S1.

Ten CCA were placed under each light treatment within each tank, yielding forty CCA per pCO_2 x light treatment. Buoyant weights of each sample were measured over the course of two days before they were placed in their experimental tanks. Samples remained in their treatments for 20-21 days, before they were weighed again at the end of the experiment. Net

calcification was calculated for each sample from its change in buoyant weight, normalized to the number of days it was exposed to experimental treatments, as well as its surface area. (As in 2014, the density of calcite was used to estimate the change in skeletal dry weight of each sample). Net photosynthesis and light-adapted (post-illumination) respiration were measured over the course of 4 days for 9 CCA from each pCO_2 x light treatment, starting after the samples had been in their experimental treatments for two weeks. Photochemical efficiency and *P-E* curve measurements were *not* made in this pilot experiment.

Results

Light had a significant effect ($p < 0.001$) on gross photosynthesis (calculated by adding the rate of oxygen consumed by respiration to the rate of oxygen evolved by net photosynthesis), causing a 46% increase in photosynthesis between the low and high light treatments. However, pCO_2 did not affect photosynthesis, nor did the interaction between pCO_2 and light (Fig. S1). In contrast, light-adapted respiration showed limited variability, increasing on average by 13% between the low and high light treatments. However, it was not significantly influenced by light, pCO_2, or the interaction of these factors (Fig S2) .

Net calcification declined by 18% in the elevated pCO_2 treatment, leading to a significant difference between the ambient and elevated treatments ($p = 0.015$). There was also a significant effect of light on calcification ($p = 0.009$), leading to a 26% increase in calcification between the low and high light treatments (Fig. S3). However, there was no significant light x pCO_2 interaction. (See Table S2 for a summary of statistical results.)

Discussion

Overall, despite differences in pCO_2 and light treatments, the results of the 2013 pilot study were qualitatively similar to the 2014 experiment. Like in 2014, light-adapted respiration

and gross photosynthesis did not respond to pCO_2 (Fig. S1, S2), but net calcification was lower in the elevated pCO_2 treatment (Fig. S3). This decline in calcification was similar in magnitude across experiments (15-18%). Additionally, in both experiments light generally had a larger effect than pCO_2, leading to a 22-26% increase in calcification, a 27-46% increase in photosynthesis, and a 13-43% increase in respiration between low and high light treatments. However, despite the positive correlation between light and photosynthesis and calcification, treatment light level did not influence the response of any of the measured physiological rates (photosynthesis, respiration, calcification) to pCO_2 (Table S2). Thus, like the 2014 experiment, light level did not modify the sensitivity of photosynthesis, respiration, or calcification to OA.

Differences between the two experiments

However, despite similarities, there were some differences between the 2013 and 2014 experiments. First, respiration did not increase significantly with light in 2013 (Table S2), unlike in 2014. This discrepancy could be explained by the smaller range of light levels used in 2013 relative to 2014 (23-210 vs. 26-413 µmol photons m^{-2} s^{-1}), and the lower sample size ($n = 9$ vs. $n = 16$), leading to lower power to detect a significant effect of light.

Additionally, the rates of respiration, photosynthesis, and calcification were higher in 2013 compared to 2014; (rates decreased on average by 5.5% in 2014 for calcification, and by 50% for respiration and photosynthesis). Several factors could explain this variation, including differences between the experiments in: 1) experimental duration, 2) tank water turnover rates, and 3) water quality. Exposure to experimental treatments was longer in 2014, potentially reducing growth and metabolic rates as the CCA acclimated to their new experimental conditions, which had lower light, flow, etc. than their natural habitat. Supporting this hypothesis, gross photosynthesis rates measured for the *P-E* curves in 2014 were generally higher (by ~ 0.1 µmol O_2 cm^{-2} hr^{-1} for the ambient pCO_2 treatment) than rates measured at

similar light levels 1-2 weeks later. Additionally, water turnover rates were higher in the experimental tanks in 2013, which also may have facilitated faster growth and metabolism in the CCA that year by increasing nutrient delivery, gas exchange, and metabolite removal. Finally, differences in water quality between the years (nutrients, pollution, etc.) that we couldn't control for with our water filtering procedure, also could have contributed to differences in physiological rates. However, no direct evidence suggests this was the case, so the first two explanations seem the most likely. Despite these differences between years, the rates we measured in both experiments were within the ranges reported for *P. onkodes* [1-5], as well as other CCA [6].

Overall, our results indicate that the effects of OA on gross photosynthesis, light-adapted respiration, and net calcification in *P. onkodes* are consistent across a range of experimental conditions, but are not necessarily larger in magnitude than the effects of other factors such as flow or light.

Tables for the 2013 pilot study

Table S1) Physical conditions of the 2013 pilot study. Values represent group means ± SEM. There were 4 tanks per pCO_2 treatment, and light treatments were nested within tanks. Treatments abbreviations are as follows—ambient pCO_2 (ACO_2) or high (HCO_2); low light (LL), medium light (ML), or light saturated, i.e., "high" light (HL). Physical variables include total alkalinity (TA), saturation state of seawater with respect to calcite ($\Omega_{calcite}$), and photon flux density (PFD) of light treatments.

**Treatment**	**Temperature**	**TA**	**pH**	**pCO_2**	**HCO_3^-**	**CO_3^{2-}**	**$\Omega_{calcite}$**	**PFD**	**Salinity**
	°C	µmol kg^{-1}		µatm	µmol kg^{-1}	µmol kg^{-1}		µmol photons m^{-2} s^{-1}	PSU
ACO_2-HL	26.9 ± 0.1	2310.4 ± 2.5	8.058 ± 0.002	380.1 ± 2.3	1732.0 ± 2.2	234.7 ± 0.9	5.62 ± .02	215.1 ± 31.7	36.0 ± 0.02
ACO_2-ML	26.9 ± 0.1	2310.4 ± 2.5	8.058 ± 0.002	380.1 ± 2.3	1732.0 ± 2.2	234.7 ± 0.9	5.62 ± .02	58.0 ± 6.1	36.0 ± 0.02
ACO_2-LL	26.9 ± 0.1	2310.4 ± 2.5	8.058 ± 0.002	380.1 ± 2.3	1732.0 ± 2.2	234.7 ± 0.9	5.62 ± .02	23.9 ± 3.2	36.0 ± 0.02
HCO_2-HL	27.3 ± 0.1	2315.4 ± 1.7	7.775 ± 0.004	832.6 ± 8.3	1967.8 ± 2.5	141.6 ± 1.0	3.39 ± .02	205.7 ± 18.6	36.1 ± 0.02
HCO_2-ML	27.3 ± 0.1	2315.4 ± 1.7	7.775 ± 0.004	832.6 ± 8.3	1967.8 ± 2.5	141.6 ± 1.0	3.39 ± .02	51.6 ± 4.8	36.1 ± 0.02
HCO_2-LL	27.3 ± 0.1	2315.4 ± 1.7	7.775 ± 0.004	832.6 ± 8.3	1967.8 ± 2.5	141.6 ± 1.0	3.39 ± .02	21.7 ± 2.7	36.1 ± 0.02

Table S2) Statistical summary of the response of various physiological rates to pCO_2 and light treatments in the 2013 experiment. Significant p-values are highlighted in bold.

Response	n	effect	df	F - statistic	p-value
Gross photosynthesis	9	pCO_2	(1, 6)	0.008	0.930
		light	(2, 41)	20.592	**< 0.0001**
		pCO_2 x light	(2, 41)	1.225	0.304
Light-adapted respiration	9	pCO_2	(1, 6)	0.211	0.662
		light	(2, 41)	2.398	0.104
		pCO_2 x light	(2, 41)	0.459	0.635
Net calcification	40	pCO_2	(1, 226)	5.987	**0.015**
		light	(2, 226)	4.817	**0.009**
		pCO_2 x light	(2, 226)	0.067	0.935

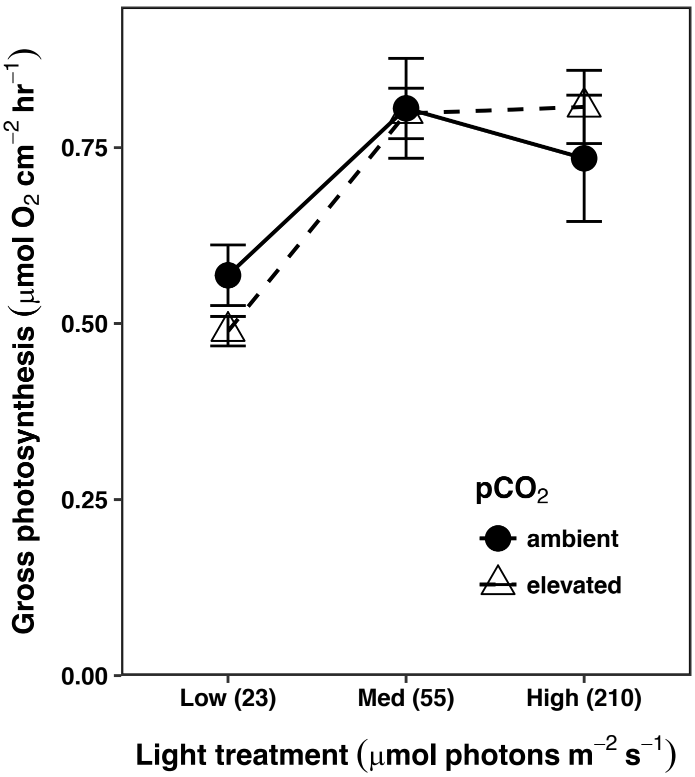

Fig. S1) Area-normalized gross photosynthesis rates ($n = 9$), measured at approximately the same photon flux density (PFD) that the CCA were kept under in their experimental light treatments. Gross photosynthesis was calculated as the sum of the rate of oxygen evolution by net photosynthesis, plus the rate of oxygen consumption by light-adapted respiration for each individual CCA sample. The x-axis indicates experimental light treatments, with the mean PFDs for each treatment in parentheses. Points represent treatment group means ± SEM. Photosynthesis increased significantly with treatment light level ($p < 0.0001$), but did not respond to pCO_2. Additionally, there was no significant light x pCO_2 interaction.

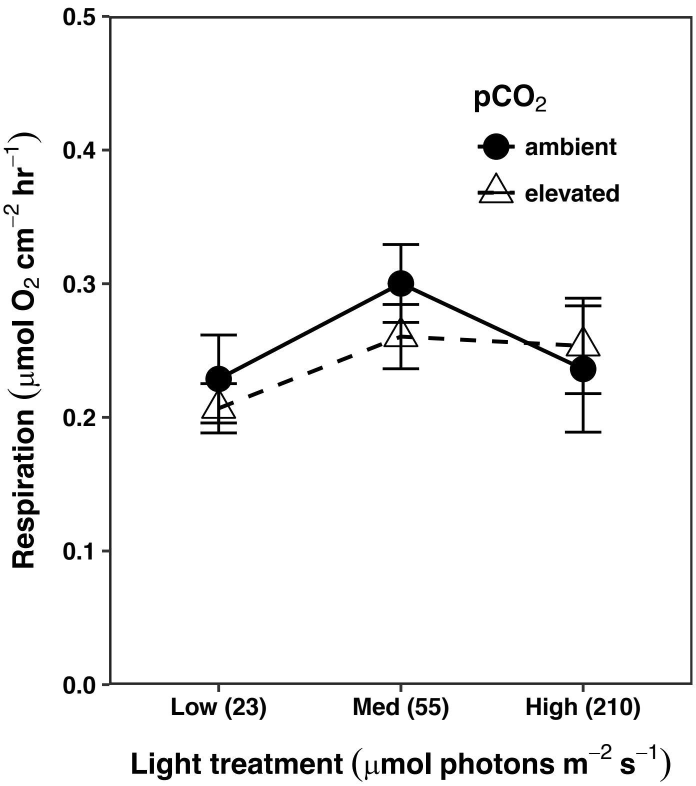

Fig. S2) Light-adapted respiration, in terms of μmol O_2 consumed per hour, normalized to the surface area of the algal samples (n = 9). Points represent treatment group means ± SEM. The x-axis indicates experimental light treatments, with the mean photon flux density (μmol photons m^{-2} s^{-1}) for each treatment in parentheses. Light and pCO_2 did not significantly affect respiration rates.

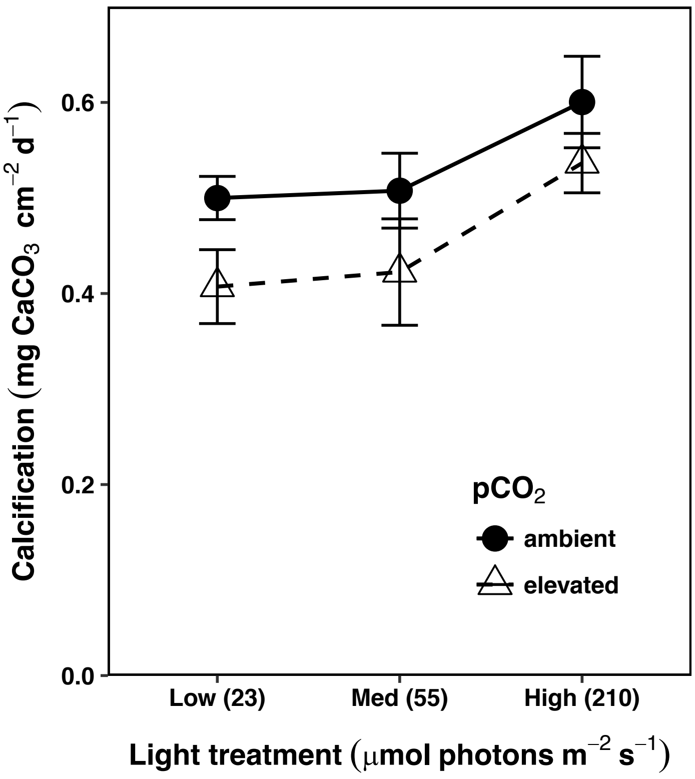

Fig. S3) Area-normalized net calcification rates of *P. onkodes*. Points represent treatment group means ± SEM (n = 40). The x-axis indicates experimental light treatments, with the mean photon flux density (μmol photons m^{-2} s^{-1}) for each treatment in parentheses. Calcification was significantly reduced by elevated pCO_2 ($p = 0.015$), and increased with light ($p = 0.009$), but there was no significant light x pCO_2 interaction.

Summary tables for the 2014 experiment

Table S3) Physical conditions of the 2014 experiment. Values represent group means ± SEM. There were 4 tanks per pCO_2 treatment, and light treatments were nested within tanks. Treatments abbreviations are as follows—ambient pCO_2 (ACO_2) or high (HCO_2); low light (LL), medium light (ML), or light saturated, i.e., "high" light (HL). Physical variables include total alkalinity (TA), saturation state of seawater with respect to calcite ($\Omega_{calcite}$), and photon flux density (PFD) of light treatments.

**Treatment**	**Temperature**	**TA**	**pH**	**pCO_2**	**HCO_3^-**	**CO_3^{2-}**	**$\Omega_{calcite}$**	**PFD**	**Salinity**
	°C	µmol kg^{-1}		µatm	µmol kg^{-1}	µmol kg^{-1}		µmol photons $m^{-2} s^{-1}$	PSU
ACO_2-HL	27.5 ± 0.1	2271.2 ± 4.3	8.029 ± .002	406.1 ± 2.3	1721.3 ± 3.2	221.1 ± 1.0	5.33 ± .02	423.5 ± 3.9	35.3 ± 0.1
ACO_2-ML	27.5 ± 0.1	2271.2 ± 4.3	8.029 ± .002	406.1 ± 2.3	1721.3 ± 3.2	221.1 ± 1.0	5.33 ± .02	70.3 ± 1.4	35.3 ± 0.1
ACO_2-LL	27.5 ± 0.1	2271.2 ± 4.3	8.029 ± .002	406.1 ± 2.3	1721.3 ± 3.2	221.1 ± 1.0	5.33 ± .02	27.3 ± 0.9	35.3 ± 0.1
HCO_2-HL	27.4 ± 0.1	2274.8 ± 3.1	7.702 ± .005	995.2 ± 15.0	1979.3 ± 4.8	120.6 ± 1.2	2.88 ± .03	402.3 ± 4.9	35.4 ± 0.1
HCO_2-ML	27.4 ± 0.1	2274.8 ± 3.1	7.702 ± .005	995.2 ± 15.0	1979.3 ± 4.8	120.6 ± 1.2	2.88 ± .03	63.4 ± 2.3	35.4 ± 0.1
HCO_2-LL	27.4 ± 0.1	2274.8 ± 3.1	7.702 ± .005	995.2 ± 15.0	1979.3 ± 4.8	120.6 ± 1.2	2.88 ± .03	24.9 ± 1.3	35.4 ± 0.1

Table S4) Statistical summary of the response of various physiological rates and P-E curve parameters to pCO_2 and light treatments in the 2014 experiment. Significant p-values are highlighted in bold.

**Response**	**n**	**effect**	**df**	**F - statistic**	**p-value**
Gross photosynthesis	16	pCO_2	(1, 6)	0.268	0.623
		light	(2, 82)	4.372	**0.016**
		pCO_2 x light	(2, 82)	2.181	0.120
Light-adapted respiration	16	pCO_2	(1, 6)	0.611	0.464
		light	(2, 84)	5.353	**0.007**
		pCO_2 x light	(2, 84)	1.348	0.265
Dark-adapted respiration	8	pCO_2	(1, 6)	4.811	0.071
		light	(2, 36)	3.209	0.052
		pCO_2 x light	(2, 36)	3.570	**0.039**
Net calcification	32	pCO_2	(1, 6)	6.298	**0.046**
		light	(2, 176)	7.135	**0.001**
		pCO_2 x light	(2, 176)	0.696	0.500
Photochemical efficiency (F_v/F_m)	32	pCO_2	(1, 6)	0.542	0.489
		light	(2, 182)	98.096	**< 0.0001**
		pCO_2 x light	(2, 182)	4.210	**0.016**
P-E curve parameters					
Initial slope (α)	8	pCO_2	(1, 6)	0.216	0.659
		light	(2, 35)	15.701	**< 0.0001**
		pCO_2 x light	(2, 35)	3.276	**0.050**
Max. gross photosynthesis (P_{max})	8	pCO_2	(1, 6)	0.207	0.665
		light	(2, 35)	0.999	0.378
		pCO_2 x light	(2, 35)	0.862	0.431

CCA identification and characterization of *in situ* light levels

Methods S1 - CCA species identification

P. onkodes crusts were identified in the field using morphological characteristics such as its thick, heavily calcified crust that strongly adhered to the substrate, its smooth, chalky epithallial texture, tight trichocyte fields, and very small to non-obvious conceptacles [7,8]. Specimens were collected to confirm identifications in the lab. Species identifications were done under the supervision of Dr. Robert S. Steneck, by analyzing specimens under a dissecting microscope. Features such as conceptacle size and shape, distribution of trichocytes, and cell shape, size, and structural arrangement (observed in cross section), allowed us to confirm the identification of the specimens as *P. onkodes*.

Since conducting this study, other researchers have determined that *P. onkodes* is a complex of greater than twenty morphologically similar species, rather than a single pantropically distributed species [9]. Since we did not preserve samples for genetic analysis, we cannot confirm the precise species identity of our CCA. However, all samples were collected from similar microhabitats (open horizontal and vertical reef substrates) at a single back reef location in Moorea, French Polynesia, suggesting they were likely a single (or very few) species. Therefore, the results of our study should be interpreted as applying to an undefined *P. "onkodes"* species, or a complex of several morphologically similar species.

Methods S2 - *In situ* light levels

Light levels characterizing the natural habitat of the experimental CCA were measured on five separate sunny days, within an hour of local apparent noon at the experimental collection site. Photon flux densities were measured at the location of *P. onkodes* crusts (or scars where crusts were recently collected), using a 2π PAR sensor attached to a diving-PAM (Walz, Germany), held parallel to the surface of the CCA. After recording the light level at a crust, the researcher swam at least 1 m in a haphazard direction until another *P. onkodes* crust was found. Light measurements were made only for *P. onkodes* in exposed reef habitats (including horizontal and vertical substrates), to remain consistent with the environment where the experimental CCA were collected. This procedure excluded some individuals that were observed in semi-cryptic microhabitats, e.g. under coral overhangs or in crevices in the reef substrate. Therefore, these measurements provide a snapshot of the light levels that the experimental CCA experienced in their natural environment, but do not characterize the full range of light environments inhabited by *P. onkodes* in the Moorean back reef.

Methods for physiology measurements

Methods S3 - Respirometry

Oxygen concentration was automatically corrected for temperature using a PreSens temperature probe, and adjusted for the average salinity of the experiment. The oxygen probe was calibrated daily using a two-point calibration of 0% and 100% oxygen, using zero-oxygen seawater and water-saturated air, respectively. Zero-oxygen seawater was produced by supersaturating seawater with sodium dithionite ($Na_2S_2O_4$). Water in the chambers was mixed at a constant rate using rotating stir bars, and was kept at an approximately constant temperature using an external circulating water bath. Samples were acclimated to the chambers for 10 minutes before their metabolic rates were measured. Measurements of photosynthesis and light-adapted respiration were made over the course of 5 days at the end of the experiment between 07:00 and 19:30 hr. Dark-adapted respiration was measured as part of the P-E curve incubations, and occurred 2 - 7 days before net photosynthesis and light-adapted respiration. A stratified random design was used to determine the order in which samples from different treatments were run, in order to eliminate the influence of incubation timing on the measured metabolic rates.

Methods S4 - *P-E* curves

P-E curve measurements began after the samples had been in their experimental treatments for two weeks, and then were made over the course of 6 days between the hours of 06:45 and 20:45 hr. As with the previously described respiration and photosynthesis measurements, a stratified random design was used to determine the order that samples from different treatments were run, to eliminate the influence of incubation timing on *P-E* curve characteristics. For each algal sample, respiration rates were measured first, after the alga had been dark-acclimated for at least 45 minutes to eliminate the stimulatory effect of photosynthesis on respiration [10]. Following respiration, incubations were conducted on the same sample at each successively increasing PFDs, allowing the sample to acclimate to the new light level for at least 10 minutes before new measurements began. For each sample, seawater in the incubation chamber was completely replaced with fresh seawater from the treatment tank after every third incubation, or after the sample had spent longer than 105 minutes in the chamber, whichever occurred first. Due to the slow metabolic rates of CCA, and based on the PreSens logger readings of oxygen concentrations, this frequency of water replacement was deemed adequate to prevent changes in the chemical environment of the chambers of large enough magnitude to influence the physiological rates of the CCA.

Methods S5 - Photochemical Efficiency (F_v / F_m)

Because of the large number of samples (n = 192), these measurements were taken over the course of four nights at the end of the experiment. Each night, algae were dark-adapted for two hours following the onset of the dark period, and then for a randomly chosen subset of samples from each light treatment in each tank, measurements were taken from a flat area in the center of each algal thallus. These measurements were made with the 5.5 mm diameter PAM probe tip positioned 0.5 cm from the algal surface using a plastic spacer. Minimum fluorescence (F_0) was measured first, and then a 0.8 second pulse of saturating actinic light was applied to the sample and maximum fluorescence (F_m) was measured. Gain settings were adjusted slightly (ranging between 7-9) when necessary between samples to maintain adequate detection of minimum fluorescence (F_0), but all other settings on the PAM (saturating width, saturating intensity, damp) were kept constant for all the measurements. These values then were used to calculate the variable fluorescence (F_v), using the equation: $F_v = (F_m – F_0)$. Maximum photochemical efficiency was calculated by dividing the variable fluorescence by the maximum fluorescence (F_v / F_m).

References

1. Johnson, M. D., Moriarty, V. W. & Carpenter, R. C. Acclimatization of the Crustose Coralline Alga Porolithon onkodes to Variable pCO2. *PLoS ONE* **9,** e87678 (2014).

2. Johnson, M. D. & Carpenter, R. C. Nitrogen enrichment offsets direct negative effects of ocean acidification on a reef-building crustose coralline alga. *Biology Letters* **14,** 20180371 (2018).

3. Anthony, K. R. N., Kline, D. I., Diaz-Pulido, G., Dove, S. & Hoegh-Guldberg, O. Ocean acidification causes bleaching and productivity loss in coral reef builders. *Proceedings of the National Academy of Sciences* **105,** 17442–17446 (2008).

4. Comeau, S. *et al.* Pacific-wide contrast highlights resistance of reef calcifiers to ocean acidification. *P Roy Soc B-Biol Sci* **281,** 20141339–20141339 (2014).

5. Chisholm, J. R. Primary productivity of reef-building crustose coralline algae. *Limnol. Oceangr.* 1376–1387 (2003).

6. Martin, S., Cohu, S., Vignot, C., Zimmerman, G. & Gattuso, J.-P. One-year experiment on the physiological response of the Mediterranean crustose coralline alga, Lithophyllum cabiochae, to elevated pCO 2and temperature. *Ecol Evol* **3,** 676–693 (2013).

7. Adey, W. H., Townsend, R. A. & Boykins, W. T. The crustose coralline algae (Rhodophyta: Corallinaceae) of the Hawaiian Islands. (1982).

8. Payri, C. E., Orempuller, J. & N'Yeurt, A. D. R. *Algues de Polynésie française = Algae of French Polynesia*. (Au Vent de Îles, Editions Tahiti, 2000).

9. Gabrielson, P. W., Hughey, J. R. & Diaz-Pulido, G. Genomics reveals abundant speciation in the coral reef building alga Porolithon onkodes(Corallinales, Rhodophyta). *Journal of Phycology* **54,** 429–434 (2018).

10. Edmunds, P. J. & Davies, P. S. Post-illumination stimulation of respiration rate in the coral Porites porites. *Coral Reefs* **7,** 7–9 (1988).
